# Oral Paraneoplastic Pemphigus: A Scoping Review on Pathogenetic Mechanisms and Histo-Serological Profile

**DOI:** 10.3390/antib13040095

**Published:** 2024-11-22

**Authors:** Domenico De Falco, Sabrina Messina, Massimo Petruzzi

**Affiliations:** Interdisciplinary Department of Medicine, University of Bari, 70124 Bari, Italy; messinasabrina96@gmail.com (S.M.); massimo.petruzzi@uniba.it (M.P.)

**Keywords:** paraneoplastic pemphigus, paraneoplastic syndromes, oral pemphigus, oral paraneoplastic syndrome, vesiculobullous disease, oral mucositis

## Abstract

Paraneoplastic pemphigus (PNP) is a rare autoimmune disorder associated with underlying neoplasms, predominantly Non-Hodgkin Lymphomas, affecting adults aged 45 to 70. This review analyzed 87 articles from MEDLINE/PubMed, Ovid and Scopus focusing on patients with oral manifestations of PNP, emphasizing histological and serological aspects and discussing recent updates on pathogenetic options. Key findings revealed that PNP is often diagnosed before the neoplasm, with Follicular variant Non-Hodgkin Lymphoma and Castleman Disease being the most common associations. Histopathological analysis showed suprabasal acantholysis and inflammation, and serological tests identify a comprehensive autoantibody panel, underscoring the need for standardized diagnostic criteria and improved serological testing.

## 1. Introduction

Paraneoplastic pemphigus (PNP) is an autoimmune mucocutaneous disorder characterized by vesiculobullous lesions, typically occurring in association with underlying malignancies [1,2,3,4,5]. This condition was first clinically identified in 1990 by Anhalt et al. In 2001, Nguyen et al. introduced the concept of paraneoplastic autoimmune multiorgan syndrome (PAMS), focusing on systemic involvement [6,7]. PNP is a rare disorder, accounting for approximately 3–5% of all pemphigus cases. It typically affects individuals between 45 and 70 years of age, with no significant gender predilection [8]. Pediatric cases of PNP, particularly those associated with Castleman Disease, are also documented [9]. In 30% of cases, PNP is the first clinical manifestation of an occult neoplasm, with oral and cutaneous manifestations often being the earliest [8]. PNP’s most frequently associated neoplasms are Non-Hodgkin’s Lymphoma (38.6%), followed by Chronic Lymphocytic Leukemia (18.4%), Castleman’s Disease (18.4%), Thymoma (5.5%), Waldenstrom’s Macroglobulinemia (1.2%), Hodgkin’s Lymphoma (0.6%) and Monoclonal Gammopathy (0.6%) [1,10,11]. PNPs secondary to carcinomas and sarcomas make up around 12% of cases [1].

PNP often presents with early oral mucosa involvement, typically manifesting, as shown in Table 1, with polymorphic erosive lesions preceded by bullous vesicles, which may lead to extensive desquamative areas, similar in morphology with pemphigus vulgaris [8,12,13]. Therefore, oral mucositis refractory to treatment may be the only presenting clinical sign of PNP [5,8,9,12]. 

These lesions can develop on different areas of the oral cavity, including tongue, palate, cheeks and lips, with a preference for the vermilion [8,13]. They are usually highly symptomatic, characterized by pain and a burning sensation [8]. Painful blisters can be debilitating, often making routine tasks such as eating, drinking, and speaking extremely difficult [4,14]. Cutaneous involvement in PNP presents a wide and heterogeneous variety of manifestations, often appearing after the onset of mucosal clinical signs. Cutaneous manifestations of PNP can include pruritic red-purple papules, targetoid plaques, diffuse erythema, flaccid blisters, and exfoliative erythroderma. Due to these clinical manifestations, PNP may resemble other mucocutaneous conditions like Erythema Multiforme, Lichen Planus, Stevens–Johnson Syndrome, Herpesvirus infections, Bullous Pemphigoid, Pemphigus Vulgaris and Graft versus Host Disease [8,15,16]. Among mucosal manifestations, ocular involvement is common and leads to dryness, pain, and corneal damage, including perforation [9]. Bronchiolitis obliterans (BO) is the most common respiratory manifestation of PNP, leading to progressive respiratory failure and often death. BO is difficult to treat with immunosuppressants, and some patients may require lung transplantation [9]. PNP can also affect the esophageal mucosa, causing erosive esophagitis and dysphagia. Additionally, patients with PNP have a higher incidence of myasthenia gravis, which may occur independently of other underlying neoplasms [9].

In 1993, Camisa and Helm created a diagnostic guide for PNP (Table 2) based on major and minor criteria. For diagnosis, three major criteria or two major and two minor criteria were required [17,18]. In 2021, a study by Svoboda and Huang proposed criteria modifications (Table 3) to increase diagnostic sensitivity, indicating that the diagnostic criteria from 1990 to 1993 failed to capture a significant portion of PNP cases [12,17,18,19].

The pathogenesis of PNP is not fully understood. It is hypothesized that the tumor condition acts as a trigger for immunological dysregulation, leading to the production of autoantibodies directed against the plakin family, including envoplakin (210 kDa), periplakin (190 kDa), bullous pemphigoid antigen I (BP230, 230 kDa), desmoplakin I (250 kDa), desmoplakin II (210 kDa), plectin (500 kDa), and α2-macroglobulin-like-1 (170 kDa) [20,21,22,23]. The literature consistently highlights the pivotal role of both autoantibodies and cell-mediated immunity in the pathogenesis of this syndrome [20,24]. PNP has a poor prognosis, with a mortality rate close to 90%, being more refractory to treatment compared to other forms of pemphigus [8,9]. Remission of the underlying neoplastic process often results in a significant improvement of patients’ paraneoplastic manifestations, which leads, in some cases, to a spontaneous resolution of the lesions [8,12]. First-line treatment includes high-dose corticosteroids, although a combination of therapies (e.g., prednisone with azathioprine or cyclosporine) is more effective. In treatment-resistant cases, monoclonal antibodies may be used, and early antibiotic therapy is recommended due to the use of immunosuppressants [8,9].

This scoping review addresses the highly varied and underexplored scientific field of pathogenetic mechanisms in PNP, a topic poorly debated in the literature. Evaluation and discussion of the proposed pathogenetic hypotheses has been provided through the analysis of the collected data. Additionally, the heterogeneity of serological findings also requires a critical interpretation, aligned with the latest research available in the literature. This review analyzes patients with oral manifestations of PNP, evaluating them based on sex/age, timing of PNP onset relative to the diagnosis of neoplasia, type of neoplasia, histological examination, and serological tests. Only studies in English with data on human patients were included, while studies on animal models and reviews were excluded. The review aims to address the main pathogenetic mechanisms and target antigens of PNP, as well as the future perspectives on guidelines and diagnostic criteria for PNP.

## 2. Materials and Methods

The present study has been registered on the Open Science Framework (OSF) portal, accessed on 11 November 2024 (https://doi.org/10.17605/OSF.IO/WYPRU). This scoping review was conducted following the Preferred Reporting Items for Systematic reviews and Meta-Analyses (PRISMA) extension for Scoping Review (File S2 and S3). For this scoping review, we included Case Reports, Clinical Conference, Clinical Study, Clinical Trial, Clinical Trial Protocol, Clinical Trial, Phase I, Clinical Trial, Phase II, Clinical Trial, Phase III, Clinical Trial, Phase IV, Comment, Controlled Clinical Trial, Letter, Multicenter Study, Observational Study, Randomized Controlled Trial, Humans, while Books chapters, Systematic Review, Review, in vitro studies, and animal models were excluded. Additionally, only studies in English were included. The study included all patients with oral manifestations of PNP, both before and after the diagnosis of neoplasm. The search was conducted using MEDLINE/PubMed, Ovid and Scopus with the search filter ‘paraneoplastic pemphigus’ oral. Articles published from 1990 to September 2024 were analyzed. A total of 107 articles were identified, of which 20 were excluded as they did not contain a diagnosis of PNP. The data from the remaining 87 articles were used to create a data table, in which patients were selected based on age, sex, timing of PNP onset relative to the diagnosis of neoplasia, neoplasia type, histological examination, and serological tests. The risk of bias of the included studies (File S4) was assessed using the CASP (Critical Appraisal Skills Programme) tools. CASP was chosen for its ability to provide a systematic evaluation of methodological quality and potential bias across various study designs. This approach ensured a standardized assessment of methodological quality.

## 3. Results

This scoping review was conducted by analyzing 87 articles retrieved from the MEDLINE/PUBMED, Ovid and Scopus database using the keywords ‘paraneoplastic pemphigus’ oral from 1990 to September 2024. Through the analysis of these studies, as seen in Table S1, a database was created in which patients were recorded based on sex/age, primary neoplasm, time of PNP onset relative to the neoplasm diagnosis, histological and serological features. Data analysis revealed that the decades of life from the first to the seventh are well represented, with no gender preference. Among the neoplasms triggering PNP, Follicular variant Non-Hodgkin Lymphomas and Castleman Disease in the Hyaline-Vascular subtype are the most represented. These are followed by Chronic Lymphocytic Leukemia and Thymoma. Other neoplasms, such as Clear Cell Renal Adenocarcinoma, Prostate Adenocarcinoma, Liver Carcinoma, Breast Adenocarcinoma, and Non-Small Cell Lung Cancer (NSCLC), are poorly represented. In a large majority of cases, the diagnosis of the neoplasm is made after the diagnosis of the paraneoplastic syndrome. The documented biopsies were performed in most cases from the oral cavity, while a smaller number were taken from the skin. Histopathological analysis of these biopsies revealed suprabasal acantholysis, sometimes intraepidermal, rarely subepidermal, with or without the presence of apoptotic and necrotic keratinocytes, and vacuolar degeneration, often accompanied by a cellular inflammatory infiltrate with a lichenoid appearance. Samples’ direct immunofluorescence was often positive, particularly in the basement membrane zone, with the detection of C3 and IgG, and rarely IgA, less frequently on the epidermal cell surface and in the intercellular space. Indirect immunofluorescence was positive in most cases on monkey esophagus or rat bladder. The analysis of patient serum, reported in Table 4, revealed the presence of a series of antibodies compatible with PNP or the underlying neoplasm, such as desmogleins 1 and 3, CA 125, vimentin, PSA, Ig γ-paraprotein, PIVKA-II, CA 15-3, and other molecules. Enzyme-Linked Immunosorbent Assay test (ELISA), where performed, evidenced an antibody profile characteristic of PNP, such as Desmoglein 1 (Dsg 1), Desmoglein 2 (Dsg 2), and Desmoglein 3 (Dsg 3), BP180, BP230, Envoplakin (EVPL), Periplakin (PPL), and Desmocollin 1, 2, and 3. Even using immunoblotting and immunoprecipitation, EVPL, PPL, Alpha 2 sub-unit Macroglobulin Like 1 (A2ML1), BP230, Dsg1, Dsg3, Desmoplakin 1, Desmoplakin 2, and Plectin were detected.

## 4. Discussion

PNP is a paraneoplastic syndrome most commonly associated with neoplasms such as Non-Hodgkin Lymphomas, Castleman Disease, Thymomas, and Leukemias [1,10]. The pathogenic mechanism is not yet fully understood; however, several studies show that both an antibody-mediated response and a cellular-mediated response are synergically involved [20,24].

In a review by Czernik et al., it is hypothesized that cell-mediated immunity may play a key role in PNP, highlighting the presence of perilesional mononuclear cells and elevated levels of IL-6 in the serum of patients with PNP [20].

According to Kim et al., the presence of lichenoid dermatitis in PNP indicates that cell-mediated immune mechanisms play an important role in its development. Additionally, the infiltration of CD8+ T cells is frequently observed in PNP, suggesting that these autoreactive T cells directly contribute to the formation of lichenoid dermatitis [26].

Lichenoid dermatitis may also be the only sign of PNP and may appear before the onset of blisters. Therefore, this suggests that lichenoid inflammation induced by cell-mediated immunity could lead to the exposure of autoantigens, such as plakins, to the immune system, thereby inducing the production of antibodies [26]. A common finding frequently reported in the literature is the complete absence of antibody components, accompanied by a predominance of CD4+ and CD8+ lymphocytes, CD56+ natural killer cells, and CD68+ macrophages, along with elevated levels of interferon-gamma and TNF-alpha across numerous samples [9,27,28]. A considerable number of these patients present a history of treatment with rituximab or other anti-CD20 therapies [29,30,31]. The underlying mechanism remains uncertain; specifically, it is unclear whether B-cell depletion merely suppresses autoantibody production or initiates an autoreactive T-cell response [30,31]. In this cohort of antibody-negative patients, cellular immunity is identified as the exclusive pathogenic factor in PNP [29,30,31,32,33,34]. Paradoxically, these patients should not be diagnosed with PNP because they do not meet the current diagnostic criteria, which require the detection of anti-plakin autoantibodies through techniques such as immunofluorescence, immunoblotting, immunoprecipitation, or ELISA. As a result, a definitive diagnosis of PNP in these patients remains a topic of ongoing debate [30,31,34].

According to Kim et al., as previously mentioned, there are several potential pathogenetic mechanisms that induce paraneoplastic autoimmunity. T cells, developing in the thymus, undergo positive and negative selections that serve to eliminate non-functional or self-reactive cells. Negative selection occurs through the expression of tissue-specific antigens by thymic medullary epithelial cells, a process mediated by the Autoimmune Regulator (Aire) [26]. However, the presence of a thymic tumor, such as a Thymoma, can compromise this crucial phase, allowing self-reactive T cells to escape central tolerance and promote autoimmune responses. Thymoma is indeed often associated with PNP and other autoimmune diseases such as myasthenia gravis. While Aire plays a determining role in this context, it is not the exclusive factor involved in regulating central tolerance in PNP [26,35]. In fact, according to the same authors, it is possible that self-reactive T cells that escape central tolerance may still be regulated by peripheral tolerance, which acts through mechanisms such as anergy, deletion, and suppression by regulatory T cells (Tregs). However, in some cases, B-cell tumors express costimulatory molecules such as CD80 and CD86, which can activate self-reactive T cells, thereby overcoming peripheral tolerance mechanisms [26]. Tregs, crucial in suppressing autoimmune responses, can, in turn, be negatively influenced by cytokines such as interleukin-6 (IL-6), which hinder their suppressive function. Elevated levels of IL-6 have been frequently observed in patients with PNP and in idiopathic multicentric Castleman disease, suggesting that an imbalance of Tregs and excess IL-6 may contribute to paraneoplastic autoimmunity in the context of PNP [26]. Ultimately, Kim et al. [26] supposed that PNP may also arise from an antitumor immune response, where tumor-specific neoantigens generated by mutations provoke a cross-reactive response of T cells with normal self-antigens, causing autoimmunity due to molecular mimicry. Although not yet investigated, neoantigens that mimic self-antigens derived from desmosomal and hemidesmosomal proteins could be relevant, considering that proteins such as Dsg3, BP180, BP230, and integrin α6β4 are overexpressed in epithelial carcinomas [26]. Once activated, the autoimmune response can cause tissue damage, promoting the activation of immune cells specific to other self-antigens through the phenomenon of epitope spreading, which explains the presence of autoantibodies against multiple self-antigens in patients with PNP [26]. The diagnosis of PNP often precedes the detection of the underlying neoplasm, representing a warning sign [5,6,36]. Diagnostic protocols involve a combination of histological examination and immunoserological findings. Although suprabasal acantholysis and lichenoid infiltrate are frequently observed, several studies indicate that these findings are often nonspecific for this condition [37]. Direct immunofluorescence sensitively detects IgG and C3, predominantly at the basement membrane [38]. However, the same findings are observed in pemphigus vulgaris or erythematosus, suggesting a low sensitivity of this diagnostic test [37,39]. Indirect immunofluorescence performed on monkey esophagus or mouse bladder shows positive results with greater sensitivity and specificity. In particular, rat bladder epithelium is preferred as a substrate [37]. Furthermore, the rat bladder is rich in plakins such as envoplakin and periplakin, making it highly specific for differentiating PNP from other forms of pemphigus [26]. ELISA is a useful technique for detecting circulating antibodies in PNP, such as those against desmogleins 1, 2, and 3, as well as desmocollins 1, 2, and 3 [21]. In a study conducted by Ohzono et al., approximately 80% of the 104 patients analyzed with PNP had circulating IgG anti-Dsg3 and other autoantibodies such as anti-Dsg1, anti-Dsc1, anti-Dsc2, and anti-Dsc3 [40]. Immunoblotting and immunoprecipitation also detect antibodies typical of PNP with extremely high sensitivity and specificity, such as anti-envoplakin and periplakin, along with other molecules like A2ML1, BP230, Dsg1, Dsg3, DSP1, DSP2, and plectin. According to the study by Tsuchisaka et al. [1], epiplakin is an autoantigen in PNP, being detected in 72.9% of their patients through immunoblotting and immunoprecipitation [1]. Detection of these molecules is the most specific diagnostic test [37].

Based on our analysis of the results and the existing literature, evidence shows that PNP often represents the first clinical condition of an underlying tumor in a significant number of cases. Typically, oral lesions are the earliest signs [5]. Given the refractory nature of these lesions to standard treatments, thorough screening for the detection of occult neoplasms becomes crucial for patients. Currently, however, no standardized protocols exist for such screenings. However, it is recommended to consider second-level instrumental exams and blood tests as mandatory examinations for all patients presenting with desquamative lesions that are resistant to treatment [6].

## 5. Conclusions

PNP is an extremely debilitating condition that primarily affects the oral cavity and skin. In a considerable number of cases, this paraneoplastic syndrome presents before the underlying neoplasm is diagnosed. However, the diagnostic process of PNP may not be straightforward, due to the similarity of its clinical manifestations (especially oral) with other conditions, most notably pemphigus vulgaris. Furthermore, as previously discussed, in many cases there is a complete absence of the antibody component, both histologically and serologically, with a predominant cellular immune response. Consequently, a modification of the diagnostic criteria and the development of procedural clinical guidelines to identify the occult neoplasms that trigger PNP is essential. The diagnostic approach for PNP typically involves a combination of clinical evaluations, histological examinations, and serological tests. Recent scientific studies have revealed new target antigens for diagnosing this condition, particularly plakin proteins. Therefore, there is a pressing need for the standardization and development of new serological tests to facilitate more straightforward and effective case identification.

## Figures and Tables

**Table 1:** Typical clinical features of PNP [9].

Polymorphism of lesions Severe and refractary lesions Treatment resistance Frequent involvement of palmar/plantar skin and ocular mucosa Poor prognosis Bronchiolitis Obliterans (BO)

**Table 2:** Diagnostic criteria from Camisa and Helm [8].

Major Criteria: Polymorphic mucocutaneous eruptions Concomitant internal neoplasm Serum antibodies with a specific immunoprecipitation pattern
Minor Criteria: Acantholysis observed histologically DIF displaying intercellular and basement membrane staining IIF staining positive on rat bladder epithelium Note: For diagnosis, 3 major criteria or 2 major and 2 minor criteria were required

**Table 3:** Svoboda and Huang modified criteria [8,12,17].

Major Criteria Mucous membrane lesions with or without cutaneous involvement Concomitant internal neoplasm Serologic evidence of anti-plakin antibodies (including but not limited to IP, IB, ELISA, IIF on transitional epithelium)
Minor Criteria Acantholysis and/or lichenoid interface dermatitis on histopathology, ± necrotic keratinocytes Direct immunofluorescence (DIF) staining showing intercellular and/or basement membrane staining.

**Table 4:** An overview of serological findings [8,9,12].

Target	Function	Associated Diseases
Desmoglein 1	Cell adhesion proteins that stabilize intercellular junctions between epithelial cells.	Pemphigus foliaceus
Desmoglein 2	Similar to Desmoglein 1, but also involved in the junctions of epidermal tissues and the skin.	Pemphigus vulgaris
Desmoglein 3	Promotes cell adhesion in the deeper layers of the epidermis and in the mucous membranes.	Pemphigus vulgaris, PNP
BP180	A protein of the dermal–epidermal junction involved in skin cohesion.	Bullous pemphigoid
BP230	A protein associated with the stability of junctions, involved in the mechanical strength of the skin.	Bullous pemphigoid, PNP
Envoplakin	Adhesion protein that stabilizes the junctions between epithelial cells.	Bullous pemphigoid, autoimmune diseases, PNP
Periplakin	Involved in stabilizing the cell matrix and maintaining the integrity of the skin.	Bullous pemphigoid, autoimmune diseases, PNP
Desmocollin 1, 2, and 3	Cell adhesion proteins, components of desmosomal junctions, important for the cohesion of epithelial cells.	Pemphigus vulgaris, Bullous pemphigoid, PNP
Alpha 2 sub-unit Macroglobulin Like 1	Binding protein that may play a role in regulating the immune response and controlling the extracellular matrix.	PNP
DSP1	Desmoplakin, a protein that acts as a link between desmosomes and the cytoskeleton of the cells.	Bullous pemphigoid, autoimmune diseases, PNP
DSP2	Protein associated with desmosomes, similar to DSP1, that aids in cellular stability.	Bullous pemphigoid, autoimmune diseases, PNP
Plectin	Protein that acts as a bridge between the cytoskeleton and other cellular structures, supporting the mechanical stability of the cells [24,25].	Junctional diseases, Ehlers–Danlos syndrome (platelet variant).

## Data Availability

All the data used in this study can be found in the “Supplementary Materials” section in Table S1.

## Supplementary Materials

The following supporting information can be downloaded at: https://www.mdpi.com/article/10.3390/antib13040095/s1, Table S1: Patients with oral manifestations of PNP; File S1: PRISMA ScR CheckList; File S2: PRISMA Flowdiagram; File S3: CASP Checklist: For Qualitative Research/Risk of Bias. References [41,42] are cited in the Supplementary Materials.
