# Supplementary material for: Oral Paraneoplastic Pemphigus: A Scoping Review on Pathogenetic Mechanisms and Histo-Serological Profile
Source: Antibodies (Basel). 2024 Nov 22;13(4):95. doi: 10.3390/antib13040095 (PMC11587122; doi:10.3390/antib13040095)
Supplement: Supplementary file 1 [file antibodies-13-00095-s001.zip › antibodies-3278957-Supplementary File S2 PRISMA Flowdiagram.pdf]

PRISMA 2020 flow diagram for new systematic reviews which included searches of databases, registers and other sources

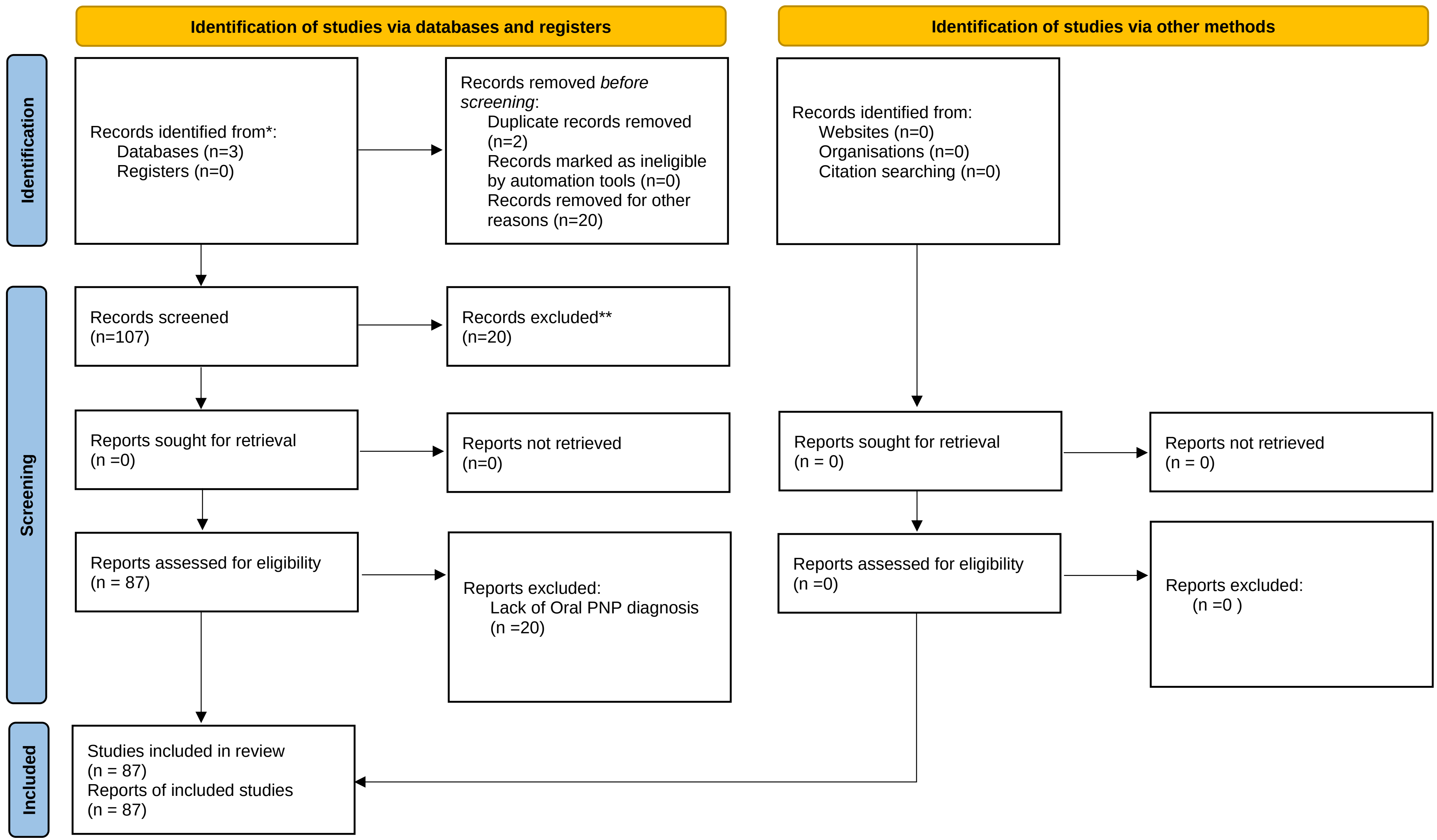

*Consider, if feasible to do so, reporting the number of records identified from each database or register searched (rather than the total number across all databases/registers).
**If automation tools were used, indicate how many records were excluded by a human and how many were excluded by automation tools.

Source: Page MJ, et al. BMJ 2021;372:n71. doi: 10.1136/bmj.n71.

This work is licensed under CC BY 4.0. To view a copy of this license, visit https://creativecommons.org/licenses/by/4.0/
