# Supplementary material for: Oral Paraneoplastic Pemphigus: A Scoping Review on Pathogenetic Mechanisms and Histo-Serological Profile
Source: Antibodies (Basel). 2024 Nov 22;13(4):95. doi: 10.3390/antib13040095 (PMC11587122; doi:10.3390/antib13040095)
Supplement: Supplementary file 1 [file antibodies-13-00095-s001.zip › antibodies-3278957-Supplementary Table S1.pdf]

Table S1. Patients with Oral Manifestations of PNP.

Authors and Country	Type	Patients	Age / Sex	Underlying neoplasm	Onset of oral manifestations, in relation to the diagnosis of neoplasia	Histopathology	Serology
**Lu SC, 2024 (Lu SC et al., 2024) Czech Republic**	Case Report	1	50/F	Tonsil invasive moderately differentiated Squamous Cell Carcinoma	Before	Subepithelial and intraepithelial blisters filled with mixed inflammatory cells dominated by neutrophils and eosinophils. Inconspicuous vacuolar alteration of the dermo-epidermal junction and exocytosis of small well-differentiated lymphocytes	**DIF**:Negative
**Ma X, 2023 (Ma X et al., 2023), China**	Case Report	1	12/F	Castleman Disease	Before	**Skin biopsy**: Suprabasal acantholysis, inflammatory cells infiltration, orthokeratosis	**IF**: NR<br>**Serum panel**: ACL, ANA, anti-nucleosome, anti-Ro/SSA, dsDNA<br>**ELISA**: NR<br>**IB**: NR<br>**IP**: NR
**Pagliaro 2023 (Pagliaro, Yau, Mortimore, & Butler, 2023), Australia**	Case Report	**1**	77 / M	Mediastinal Thymoma	Before	**Oral biopsy**: lichenoid inflammatory pattern with a dermoepidermal junction lymphocytic infiltrate and apoptotic bodies	**IF**: negative<br>**Serum panel**: NR<br>**ELISA**: NR<br>**IB**: NR<br>**IP**: NR
**Irrera 2023 (Irrera et al., 2023), Italy**	Case Report	1	13/F	Retroperitoneal Multicentric Castleman Disease-Hyalin Vascular type	Before	**Skin biopsy**: epidermal hyperplasia, spongiosis and lymphocytic infiltrate in the upper dermis.	**DIF**: C3 and IgG ICS<br>**IIF**: positive on ME<br>**Serum panel**: Dsg1, Dsg3<br>**ELISA**: NR<br>**IB**: NR<br>**IP**: NR
**Barry KK, Plumptre I, Bazewicz CG, Green BP, Treat JR, Hughes M,**	Multicentric study	1	**12/F**	Retroperitoneal Multicentric Castleman Disease-Hyalin Vascular type	Before		**IIF**: positive on ME and RBE<br>**Serum panel**: NR<br>**ELISA**: Dsg1, Dsg3

**Morel KD, Wiss K, Liang MG. 2023, US**							
		1	10 / M	Axillary Unicentric Castleman Disease-Hyalin Vascular type	Before		**IIF**: positive on ME and RBE<br>**Serum panel**: NR<br>**ELISA**: BP180, Dsg1, Dsg3<br>**IB**: NR<br>**IP**: NR
		1	7 / F	Retroperitoneal Unicentric Castleman Disease-Hyalin Vascular type	Before		**IIF**: positive on ME and RBE<br>**Serum panel**: AChR<br>**ELISA**: Dsg3<br>**IB**: NR<br>**IP**: NR
		1	8 / M	Retroperitoneal Unicentric Castleman Disease-Hyalin Vascular type	Before		**IIF**: positive on ME and RBE<br>**Serum panel**: NR<br>**ELISA**: Dsg1, Dsg3<br>**IB**: NR<br>**IP**: NR
**Gambichler 2022 (Gambichler et al., 2022), Germany**	Case report	1	70 / M	Stage IV Follicular Non-Hodgkin Lymphoma	Before	**Skin biopsy**: dyskeratosis, inflammatory cells infiltration, necrotic keratinocytes, and vacuolar Interface Dermatitis	**DIF**: negative<br>**IIF**: negative<br>**Serum panel**: NR<br>**ELISA**: negative<br>**IB**: NR<br>**IP**: NR
**Zhang 2021 (H. Zhang et al., 2021), China**	Case report	1	39 / F	Mediastinal type A Thymoma	Before	**Skin biopsy**: Intraepidermal acantholysis, inflammatory cells infiltration, and vacuolar degeneration	**DIF**: reticular C3, IgA, and IgG at BMZ<br>**IIF**: positive on RBE<br>**Serum panel**: NR<br>**ELISA**: Dsg1, Dsg3<br>**IB**: NR<br>**IP**: NR

**Liska 2022 (Liska et al., 2022), Czech Republic**	Case report	1	50 / F	Tonsillar Squamous Cell Carcinoma	Before	**Oral biopsy**: Intraepidermal and Subepidermal acantholysis and inflammatory cells infiltration	**DIF**: negative<br>**IIF**: NR<br>**Serum panel**: NR<br>**ELISA**: NR<br>**IB**: NR<br>**IP**: NR
**Poonia 2021 (Poonia, Chabra, Dalal, & Bhalla, 2021), India**	Case report	1	35 / F	Mediastinal B2-type Thymoma	Before	**Skin biopsy**: Suprabasal acantholysis and apoptotic keratinocytes	**DIF**: C3 at BMZ and IgG ICS<br>**IIF**: NR<br>**Serum panel**: NR<br>**ELISA**: NR<br>**IB**: NR<br>**IP**: NR
**Higgins 2021 (Higgins & West, 2021), US**	Case report	1	54 / M	Stage IV Follicular Non-Hodgkin Lymphoma	Before		
**Chen 2020 (X. Chen, Fu, Yang, & Li, 2020), China**	Case report	1	48 / F	Mediastinal Follicular Dendritic Cell Sarcoma with Lymph Nodes Metastasis	Before		
**Shirai 2020 (Shirai et al., 2020), Japan**	Case report	1	74 / M	Waldenström Macroglobulinemia	After	**Oral biopsy**: vacuolar Interface dermatitis	**DIF**: IgG ECS<br>**IIF**: positive<br>**Serum panel**: Dsg1, Dsg3<br>**ELISA**: Dsc2, Dsc3<br>**IB**: EVPL, PPL<br>**IP**: NR
**Wang 2019 (J. Wang, Wang, Xu, & Song, 2019), China**	Case report	1	56 / F	Retroperitoneal Follicular Dendritic Cell Sarcoma with	Before	**Skin biopsy**: Intraepidermal acantholysis, inflammatory cells infiltration, necrotic keratinocytes, and vacuolar degeneration	**DIF**: IgG ICS<br>**IIF**: positive on RBE<br>**Serum panel**: NR<br>**ELISA**: Dsg3

				Castleman Disease-Hyalin Vascular type			**IB**: Dsg3, EVPL, PPL<br>**IP**: NR
**Xiao 2019 (Xiao, Zhu, & Li, 2019), China**	Case report	1	18 / M	Retroperitoneal Castleman Disease-Hyalin Vascular type	Before		
**Lu 2019 (Lu et al., 2019), China**	Case report	1	49 / F	Pancreatic Follicular Dendritic Cell Sarcoma	Before	**Biopsy**: NR	**IF**: NR<br>**Serum panel**: CENP-B, ANA, ANCA, AChR, CA 125<br>**ELISA**: NR<br>**IB**: NR<br>**IP**: NR
**Han 2018 (Han, Fu, & Chen, 2018), Taiwan**	Case report	1	13 / F	Retroperitoneal Castleman Disease-Hyalin Vascular type Stroma rich type	Before	**Oral biopsy**: Suprabasal acantholysis and inflammatory cells infiltration	**DIF**: negative<br>**IIF**: positive on ME<br>**Serum panel**: ANA<br>**ELISA**: NR<br>**IB**: NR<br>**IP**: NR
**Okahashi 2018 (Okahashi et al., 2018), Japan**	Case report	1	68 / M	Mediastinal Thymoma with Lymph Node Metastasis	After	**Skin biopsy**: acanthosis, cytoid bodies, inflammatory cells infiltration, and vacuolar degeneration	**DIF**: C3 and IgG ECS<br>**IIF**: positive on human skin and RBE<br>**Serum panel**: NR<br>**ELISA**: BP230, Dsg3<br>**IB**: BP230, EVPL, PPL<br>**IP**: NR
**Decaux 2018 (Decaux, Ferreira, Van Eeckhout, Dachelet, & Magremanne, 2018), Belgium**	Case report	1	78 / M	Stage B B Cell Chronic Lymphocytic Leukemia	After	**Oral biopsy**: Suprabasal acantholysis, inflammatory cells infiltration, lichenoid dermatitis, and necrotic keratinocytes	**DIF**: C3 and IgG ICS<br>**IIF**: positive on RBE<br>**Serum panel**: NR<br>**ELISA**: Dsg3<br>**IB**: NR<br>**IP**: NR
**Flood 2018 (Flood, Stroud, Lazarova, Vigneswaran, & Hsu, 2018), US**	Case report	1	50 / F	Extranodal Uterine Follicular Dendritic Cell Sarcoma	Before	**Oral biopsy**: Suprabasal acantholysis, inflammatory cells infiltration, and parakeratosis	**DIF**: IgG ICS<br>**IIF**: positive on ME and RBE<br>**Serum panel**: NR<br>**ELISA**: EVLP, PPL<br>**IB**: EVLP, PPL<br>**IP**: NR

**Fu 2018 (Fu, Liu, Chen, Yang, & Li, 2018), China**	Case report	1	36 / F	Retroperitoneal Castleman Disease-Mixed Type	Before	**Biopsy**: NR	**DIF**: NR<br>**IIF**: positive on RBE<br>**Serum panel**: NR<br>**ELISA**: NR<br>**IB**: NR<br>**IP**: NR
**Balighi 2017 (Balighi, Azizpour, Sadeghinia, & Saeidi, 2017), Iran**	Case report	1	25 / M	Low-Grade Retroperitoneal Inflammatory Myofibroblastic Tumour	Before	**Skin biopsy**: Suprabasal and Subepidermal acantholysis, apoptotic keratinocytes, inflammatory cells infiltration, and vacuolar degeneration	**DIF**: granular C3 at BMZ, IgG ICS<br>**IIF**: positive on RBE<br>**Serum panel**: Vimentin, α-SMA<br>**ELISA**: Dsg1, Dsg3<br>**IB**: NR<br>**IP**: NR
**Kikuchi 2017 (Kikuchi et al., 2017), Japan**	Case report	1	74 / F	Grade II Follicular Non-Hodgkin Lymphoma with Recurrence	After	**Oral biopsy:** Suprabasal acantholysis and vacuolar degeneration	**DIF**: NR<br>**Serum panel**: NR<br>**ELISA**: NR<br>**IB**: NR<br>**IP**: NR
**Abid 2018 (Abid et al., 2018), UK**	Case report	1	46 / M	Mediastinal Unicentric Castleman Disease-Hyalin Vascular type	After	NR	
**Okahashi 2017 (Okahashi et al., 2017), Japan**	Case report	1	41 / M	Abdominal Unicentric Castleman Disease	Before	**Oral biopsy**: Suprabasal acantholysis, acanthosis, Cytoid bodies, inflammatory cells infiltration, and vacuolar degeneration	**DIF**: linear C3 at BMZ, C3 and IgG ECS<br>**IIF**: positive on human skin and RBE<br>**Serum panel**: AChR<br>**ELISA**: Dsg1, Dsg3<br>**IB**: EVPL, PPL<br>**IP**: NR
**Piscopo 2018 (Piscopo, Romano, Maria, Vinciguerra, & Vinciguerra, 2018), Italy**	Case report	1	77 / M	Prostatic Adenocarcinoma	Before	**Oral biopsy**: Suprabasal acantholysis and inflammatory cells infiltration	**DIF**: C3 and IgG ICS<br>**IIF**: positive<br>**Serum panel**: PSA, Dsg1, Dsg3<br>**ELISA**: NR<br>**IB**: Dsg1, Dsg3, DSP1, DSP2, PPL<br>**IP**: NR

**Maier 2017 (Maier et al., 2017), Austria**	Case report	1	54 / M	Epigastric Castleman Disease-Hyalin Vascular type	Before	**Oral biopsy**: Suprabasal acantholysis, dyskeratosis, and inflammatory cells infiltration	**DIF**: Fibrinogen at BMZ, C3 ICS **IIF**: positive on RBE **Serum panel**: NR **ELISA**: BP180 **IB**: negative **IP**: NR
**Oh 2016 (Oh, Lee, Hashimoto, & Kim, 2016), Korea**	Case report	1	57 / F	Retroperitoneal Castleman Disease-Hyalin Vascular type	Before	**Skin biopsy**: Suprabasal acantholysis	**DIF**: C3 and IgG at BMZ, IgG ECS **IIF**: positive on human skin and RBE **Serum panel**: NR **ELISA**: BP180, Dsc1, Dsc2, Dsc3, Dsg1, Dsg3 **IB**: BP180, EVPL, LAD-1 protein, p200, PPL **IP**: NR
**Otsuka 2016 (Otsuka et al., 2016), Japan**	Case report	1	63 / M	Non-Hodgkin Lymphoma B cell Lymphoma type	After	**Skin biopsy**: necrotic keratinocytes and vacuolar Interface dermatitis	**DIF**: C3 at BMZ, C3 and IgG ECS **IIF**: positive on ME and RBE **Serum panel**: NR **ELISA**: Dsg1, Dsg3 **IB**: BP180, BP230, Dsg3, EVPL, PPL **IP**: NR
**Wang 2016 (L. Wang, Deng, & Mao, 2016), China**	Case report	1	60 / F	Mediastinal Inflammatory pseudotumor-like Follicular Dendritic cell Sarcoma	After		
**Dhull 2016 (Dhull et al., 2016), India**	Case report	1	30 / F	Retroperitoneal Inflammatory Myofibroblastic Tumour with Bone Metastasis	Before	**Skin biopsy**: NR	**DIF**: positive **IIF**: NR **Serum panel**: NR **ELISA**: NR **IB**: NR **IP**: NR

**Namba 2016 (Namba et al., 2016), Japan**	Case report	1	59 / F	Non-Hodgkin Lymphoma B cell Lymphoma type	Before	**Skin biopsy**: apoptotic keratinocytes, inflammatory cells infiltration, and vacuolar degeneration	**DIF**: C3 at BMZ, C3 and IgG ICS **IIF**: positive on human skin and RBE **Serum panel**: NR **ELISA**: Dsg3 **IB**: EVPL, PPL **IP**: NR
**Healy 2015 (Healy, Peters, & Nana-Sinkam, 2015), US**	Case report	1	51 / M	Pelvic Castleman Disease-Hyalin Vascular type	Before	**Oral biopsy**: Suprabasal acantholysis and necrotic keratinocytes	**DIF**: C3, C5, and IgG intracellular staining **IIF**: NR **Serum panel**: ANA, dsDNA, Dsg1, Dsg3 **ELISA**: NR **IB**: NR **IP**: NR
**Kelly 2015 (Kelly, Schifter, Fulcher, & Lin, 2015), Australia**	Case series	1	34 / M	Pelvic Castleman Disease-Hyalin Vascular type Sero-rich type	Before	**Oral biopsy**: Suprabasal acantholysis and lichenoid infiltrate	**DIF**: linear C3, fibrinogen, and IgG at BMZ, C3 and IgG ICS **IIF**: positive on RBE **Serum panel**: NR **ELISA**: NR **IB**: EVPL **IP**: NR
**Ghandi 2015 (Ghandi et al., 2015), Iran**	Case report	1	30 / F	Mediastinal Inflammatory Myofibroblastic Tumour	Before	**Skin biopsy**: Intraepidermal and Suprabasal acantholysis, apoptotic keratinocytes, dyskeratosis, inflammatory cells infiltration, and melanin incontinence	**DIF**: linear C3 and IgG at BMZ, C3 and IgG ECS **IIF**: positive on RBE **Serum panel**: NR **ELISA**: Dsg1, Dsg3 **IB**: NR **IP**: NR
**Al Zamel 2015 (Al Zamel, Micheletti, Nasta, Palakshappa, & Stoopler, 2015), US**	Case report	1	55 / M	Follicular Non-Hodgkin Lymphoma	Before	**Oral biopsy**: non-specific ulcers	**DIF**: negative **IIF**: positive on ME **Serum panel**: NR **ELISA**: Dsg3 **IB**: NR **IP**: NR
**Kanaoka 2014 (Kanaoka et al., 2014),**	Case report	1	64 / F	Grade I	Before	**Skin biopsy**: Suprabasal acantholysis and Interface dermatitis	**DIF**: IgG ECS **IIF**: positive on RBE

**Japan**				Follicular Non-Hodgkin Lymphoma			**Serum panel**: NR<br>**ELISA**: BP180, Dsg3<br>**IB**: EVPL, PPL<br>**IP**: NR
**Gissi 2013 (Gissi, Bernardi, D'Andrea, & Montebugnoli, 2013), Italy**	Case report	1	79 / M	monoclonal gammopathy of unknown significance	Before	**Oral biopsy**: Intraepidermal acantholysis, hyperplasia, inflammatory cells infiltration, and necrotic keratinocytes	**DIF**: C3, fibrinogen, and IgG at BMZ; C3, fibrinogen, and IgG ICS<br>**IIF**: positive on RBE<br>**Serum panel**: Ig γ-paraprotein<br>**ELISA**: NR<br>**IB**: NR<br>**IP**: NR
**Ishii 2012 (N. Ishii et al., 2012), Japan**	Case report	1	79 / F	Mediastinal Thymoma	Before	**Skin biopsy**: Suprabasal acantholysis, hyperkeratosis, inflammatory cells infiltration, and necrotic keratinocytes	**DIF**: C3 and IgG ECS<br>**IIF**: positive on human skin and RBE<br>**Serum panel**: NR<br>**ELISA**: Dsg3<br>**IB**: Dsg3, EVPL, PPL<br>**IP**: NR
**Iida 2012 (Iida et al., 2012), Japan**	Case report	1	44 / F	Pelvic Castleman Disease-Hyalin Vascular type	Before	**Oral biopsy**: acantholysis, inflammatory cells infiltration, and vacuolar degeneration	**DIF**: linear C3 at BMZ, C3 ECS<br>**IIF**: positive on RBE<br>**Serum panel**: NR<br>**ELISA**: Dsg3<br>**IB**: EVPL, PPL<br>**IP**: NR
**Yan 2010 (Z. Yan, Hua, & Gao, 2010), China**	Case series	1	65 / M	Chronic lymphocytic leukaemia	After	**Oral biopsy**: inflammatory cells infiltration and vacuolar degeneration	**DIF**: IgG ICS<br>**IIF**: positive on RBE<br>**Serum panel**: NR<br>**ELISA**: Dsg1, Dsg3<br>**IB**: NR<br>**IP**: NR
**Ahuero 2010 (Ahuero et al., 2010) , US**	Case series	1	54 / M	B cell Chronic lymphocytic leukaemia	After	**Skin biopsy**: Subepidermal acantholysis and inflammatory cells infiltration	**DIF**: linear/granular C3 and IgG at BMZ; IgG ICS<br>**IIF**: positive on RBE<br>**Serum panel**: NR<br>**ELISA**: NR

							**IB**: NR<br>**IP**: DSP1, DSP2, EVPL, PPL
**Miltenyi 2009 (Miltenyi et al., 2009), Hungary**	Case report	1	30 / F	Pelvic Castleman Disease-Hyalin Vascular type	After	**Oral biopsy**: acantholysis, inflammatory cells infiltration, and intercellular oedema	**DIF**: C3 and IgG ICS<br>**IIF**: positive<br>**Serum panel**: NR<br>**ELISA**: NR<br>**IB**: NR<br>**IP**: NR
**Batista 2008 (Batista et al., 2008), Brazil**	Case report	1	55 / M	Non-Hodgkin Lymphoma Diffuse Large B-cell Lymphoma type	Before	**Skin biopsy**: acantholysis	**DIF**: linear C3 at BMZ, IgG ICS<br>**IIF**: positive on RBE<br>**Serum panel**: NR<br>**ELISA**: NR<br>**IB**: NR<br>**IP**: NR
**Lee 2008 (S. E. Lee, Kim, Hashimoto, & Kim, 2008), Japan**	Case report	1	67 / M	Mediastinal Thymoma and Abdominal Follicular Dendritic Cell Sarcoma with Liver Metastasis	After	**Oral biopsy**: Suprabasal acantholysis and vacuolar degeneration	**DIF**: C3 and IgG at BMZ<br>**IIF**: positive on human skin<br>**Serum panel**: NR<br>**ELISA**: negative<br>**IB**: EVPL, PPL<br>**IP**: NR
**Utsman 2008 (Utsman, Epstein, & Elad, 2008), Israel**	Case report	1	59 / M	Follicular Non-Hodgkin Lymphoma	After	**Oral biopsy**: Suprabasal acantholysis, inflammatory cells infiltration, spongiosis, and vacuolar degeneration	**IF**: NR<br>**Serum panel**: NR<br>**ELISA**: NR<br>**IB**: NR<br>**IP**: NR
**Barnadas 2006 (Barnadas et al., 2009), Spain**	Case Series	1	60/M	Stage IV Follicular Non-Hodgkin Lymphoma		Lichenoid dermatitis associated with acantholysis	**DIF: IgG**<br>**IIF: positive on RBE and human skin**<br>**IP: 210, 190**
		1	77/F	Stage IIB Follicular Non-Hodgkin Lymphoma		Lichenoid dermatitis	**IIF: positive on RBE and human skin**<br>**IB: 250,230, 210**
		1	58/M	Stage IVB, Grade I, Follicular Lymphoma		Lichenoid dermatitis plus interphase dermatitis around sweat ducts and hair follicles	**IIF: positive on RBE and human skin**

							**IB: 250,230, 210, 130, 95**
**Wang 2007 (S. H. Wang et al., 2007), Taiwan**	Case report	1	44 / F	Spleen Non-Hodgkin Lymphoma Diffuse small cleaved cell lymphoma type	Before	**Skin biopsy**: Suprabasal acantholysis, lichenoid infiltrate, and vacuolar degeneration	**DIF**: granular C3 at BMZ, C1q and IgG ICS<br>**IIF**: NR<br>**Serum panel**: NR<br>**ELISA**: NR<br>**IB**: NR<br>**IP**: NR
**Lee 2007 (D. H. Lee, Lee, & Sung, 2007), Korea**	Case report	1	48 / F	Inflammatory myofibroblastic tumour	Before	**Oral biopsy**: NR	**DIF**: C3 and IgG ICS<br>**IIF**: NR<br>**Serum panel**: NR<br>**ELISA**: NR<br>**IB**: NR<br>**IP**: NR
**Fukumoto 2007 (Fukumoto et al., 2007), Japan**	Case report	1	64 / F	Low-grade Lymphoma	Before	**Skin biopsy**: Subepidermal acantholysis, lichenoid Interface dermatitis, necrotic keratinocytes, and vacuolar degeneration	**DIF**: IgG ECS<br>**IIF**: positive on human skin and RBE<br>**Serum panel**: NR<br>**ELISA**: Dsg1<br>**IB**: EVPL, PPL<br>**IP**: NR
**Duparc 2006 (Duparc, Boivin, Gilbert, Piette, & Delaporte, 2006), France**	Case report	1	54 / F	Retroperitoneal Non-Hodgkin Lymphoma Diffuse large B-cell lymphoma type	Before	**Skin biopsy**: Suprabasal acantholysis, acanthosis, inflammatory cells infiltration, and spongiosis	**DIF**: IgG ECS<br>**IIF**: positive on RBE<br>**Serum panel**: NR<br>**ELISA**: Dsg3<br>**IB**: BP230, Dsg3, EVPL, PPL<br>**IP**: NR
**Yokokura 2006 (Yokokura et al., 2006), Japan**	Case report	1	58 / M	Hepatocellular Carcinoma	Before	**Extraoral mucosa biopsy**: Intra epidermal and Suprabasal acantholysis, inflammatory cells infiltration, and necrotic keratinocytes	**DIF**: linear fibrinogen at BMZ and IgG ECS<br>**IIF**: positive on human skin, negative on RBE<br>**Serum panel**: AFP, PIVKA-II<br>**ELISA**: Dsg3<br>**IB**: Dsg3, EVPL, PPL<br>**IP**: NR

**Lee 2006 (J. S. S. Lee, Ng, Tao, & Lim, 2006), Singapore**	Case report	1	69 / M	Stage III A Non-Hodgkin Lymphoma Follicular small cleaved cell lymphoma type	After	**Skin biopsy**: Suprabasal acantholysis, apoptotic keratinocytes, necrotic keratinocytes, and inflammatory cells infiltration	**DIF**: C3 and IgG at BMZ, C3 and IgG ICS<br>**IIF**: positive on ME and RBE<br>**Serum panel**: NR<br>**ELISA**: NR<br>**IB**: NR<br>**IP**: Dsg1, EVPL, PPL
**Barnadas 2006 (Barnadas et al., 2006), Spain**	Case report	1	77 / F	Stage IIB Follicular Non-Hodgkin Lymphoma	After	**Skin biopsy**: lichenoid Interface dermatitis, parakeratosis, and vacuolar degeneration	**DIF**: C3 and IgG at BMZ, C3 and IgG ICS<br>**IIF**: positive on ME and RBE<br>**Serum panel**: NR<br>**ELISA**: NR<br>**IB**: NR<br>**IP**: NR
**Tilakaratne 2005 (Tilakaratne & Dissanayake, 2005), Sri Lanka**	Case report	1	29 / F	Non-Hodgkin Lymphoma	After	**Skin biopsy**: Intra epidermal acantholysis, inflammatory cells infiltration, Interface dermatitis, and necrotic keratinocytes	**DIF**: IgG ICS<br>**IIF**: NR<br>**Serum panel**: NR<br>**ELISA**: NR<br>**IB**: NR<br>**IP**: NR
**Martínez De Pablo 2005 (Martínez De Pablo et al., 2005), Spain**	Case report	1	70 / F	Stage IVA Follicular Non-Hodgkin Lymphoma with recurrence and Bone Marrow Metastasis	After	**Skin biopsy**: Intraepidermal and Suprabasal acantholysis	**DIF**: granular IgG at BMZ, C3 and IgG ICS<br>**IIF**: positive on human skin, ME, and RBE<br>**Serum panel**: NR<br>**ELISA**: NR<br>**IB**: NR<br>**IP**: EVPL, PPL

**Coelho 2005 (Coelho, Reis, Tellechea, Figueiredo, & Black, 2005), Portugal**	Case report	1	74 / F	Low-Grade Pararenal Non-Hodgkin Lymphoma B cell Lymphoma type	Before	**Skin biopsy**: Interface dermatitis and vacuolar degeneration	**DIF**: focal C3 at BMZ, cytoid bodies, and IgG ICS<br>**IIF**: positive on human skin, ME, and RBE<br>**Serum panel**: ANA<br>**ELISA**: NR<br>**IB**: NR<br>**IP**: DSP1, Dsg3, EVPL, PPL
**Seishima 2004 (Seishima et al., 2004), Japan**	Case report	1	64 / F	Retroperitoneal Follicular Dendritic cell Sarcoma with Recurrence	After	**Oral biopsy**: subepidermal acantholysis and inflammatory cells infiltration	**DIF**: IgG ECS<br>**IIF**: positive on human skin and RBE<br>**Serum panel**: NR<br>**ELISA**: Dsg1, Dsg3<br>**IB**: EVPL, PPL<br>**IP**: NR
**Van Rossum 2004 (Van Rossum et al., 2004), Netherlands**	Case report	1	70 / M	Stage IVB Metastatic Follicular Non-Hodgkin Lymphoma	After	**Skin biopsy**: blister-forming dermatosis	**DIF**: linear C3 and fibrin at BMZ, C3 and IgG ECS<br>**IIF**: NR<br>**Serum panel**: NR<br>**ELISA**: Dsg3<br>**IB**: DSP, EVPL, PPL<br>**IP**: DSP, EVPL, PPL
**Preisz 2004 (Preisz et al., 2004), Hungary**	Case report	1	48 / F	Poorly Differentiated Grade III Non-Hodgkin Lymphoma	After	**Skin biopsy**: Suprabasal acantholysis, dyskeratosis, Interface dermatitis, inflammatory cells infiltration, necrotic keratinocytes, and vacuolar degeneration	**DIF**: linear C3 at BMZ; C3, IgA, and IgG ECS<br>**IIF**: positive on ME and RBE<br>**Serum panel**: NR<br>**ELISA**: Dsc3, Dsg3<br>**IB**: BP180, EVPL, PPL<br>**IP**: NR
**Powell 2004 (Powell et al., 2004), UK**	Case report	1	69 / M	Chronic Lymphocytic Leukaemia	After	**Skin biopsy**: Suprabasal acantholysis and spongiosis	**DIF**: C3 and IgG at BMZ, C3 and IgG ICS<br>**IIF**: positive on human skin, ME, and RBE<br>**Serum panel**: NR<br>**ELISA**: Dsg1, Dsg3

							**IB**: α chain of type VII collagen, A2ML1, Dsg3, DSP1, DSP2, PPL<br>**IP**: NR
**Vun 2004 (Vun, Lun, & Strutton, 2004), Australia**	Case report	1	77 / F	Retroperitoneal Non-Hodgkin Lymphoma	After	**Skin biopsy**: Suprabasal acantholysis and lichenoid Interface dermatitis	**DIF**: linear C3 at BMZ, C3 and IgG ICS<br>**IIF**: positive on ME and RBE<br>**Serum panel**: NR<br>**ELISA**: NR<br>**IB**: NR<br>**IP**: NR
**Kaplan 2004 (Kaplan et al., 2004), Israel**	Case report	1	62 / F	Breast Adenocarcinoma with Lymph Node Metastasis	Before	**Oral biopsy**: Intraepidermal acantholysis and Interface dermatitis	**DIF**: C3 and IgG at BMZ, C3 and IgG ICS<br>**IIF**: positive on RBE<br>**Serum panel**: CA 15-3<br>**ELISA**: NR<br>**IB**: NR<br>**IP**: positive
**Gergely 2003 (Gergely, Váróczy, Vadász, Remenyik, & Illés, 2003), Hungary**	Case report	1	52 / M	Stage IVB Low-Grade Non-Hodgkin Lymphoma Diffuse large B-cell lymphoma type	After	NR	
**Aessopos 2003 (Aessopos et al., 2003), Greece**	Case report	1	72 / F	Stage II Claear Cell Renal cell carcinoma	Before	**Oral biopsy**: Intraepidermal and Suprabasal acantholysis, dyskeratosis, inflammatory cells infiltration, lichenoid Interface dermatitis, necrotic keratinocytes, spongiosis, and vacuolar degeneration	**DIF**: C3 and IgG at BMZ, C3 and IgG ICS<br>**IIF**: negative on rat oesophagus<br>**Serum panel**: NR<br>**ELISA**: NR<br>**IB**: NR<br>**IP**: NR
**Mimouni 2002 (Mimouni et al., 2002), Spain**	Retrospective cohort	1	9 / M	Retroperitoneal	Before		**DIF**: negative<br>**IIF**: positive on ME and RBE<br>**Serum panel**: NR

			Unicentric Castleman disease mixed type		**Skin biopsy**: inflammatory cells infiltration, lichenoid features, and vacuolar degeneration	**ELISA**: Dsg3<br>**IB**: Plectin<br>**IP**: A2ML1, DSP1, DSP2
	1	11 / F	Castleman disease hyaline vascular type	Before	**Skin biopsy**: Intraepidermal and Subepidermal acantholysis, inflammatory cells infiltration, lichenoid features, and vacuolar degeneration	**DIF**: C3 at BMZ, C3 and IgG ICS<br>**IIF**: positive on ME and RBE<br>**Serum panel**: NR<br>**ELISA**: Dsg3<br>**IB**: Plectin<br>**IP**: A2ML1, DSP1, DSP2
	1	12 / M	Sarcoma	Before	**Oral biopsy**: Intraepidermal acantholysis and vacuolar degeneration	**DIF**: C3 at BMZ, C3 and IgG ICS<br>**IIF**: positive on ME and RBE<br>**Serum panel**: NR<br>**ELISA**: negative<br>**IB**: Plectin<br>**IP**: A2ML1, DSP1, DSP2
	1	13 / F	Castleman disease hyaline vascular type	Before	**Skin biopsy**: Intraepidermal acantholysis and lichenoid features	**DIF**: C3 and IgG ICS<br>**IIF**: positive on ME and RBE<br>**Serum panel**: NR<br>**ELISA**: Dsg1, Dsg3<br>**IB**: Plectin<br>**IP**: A2ML1, DSP1, DSP2
	1	15 / F	Castleman disease hyaline vascular type	Before	**Skin biopsy**: inflammatory cells infiltration, lichenoid features, and vacuolar degeneration	**DIF**: C3 and IgG at BMZ, C3 and IgG ICS<br>**IIF**: positive on ME and RBE<br>**Serum panel**: NR

						**ELISA**: Dsg1, Dsg3<br>**IB**: Plectin<br>**IP**: A2ML1, DSP1, DSP2
	1	16 / F	Castleman disease hyaline vascular type	Before	**Oral biopsy**: inflammatory cells infiltration, lichenoid features, and vacuolar degeneration	**DIF**: C3 and IgG ICS<br>**IIF**: positive on ME and RBE<br>**Serum panel**: NR<br>**ELISA**: negative<br>**IB**: Plectin<br>**IP**: A2ML1, DSP1, DSP2
	1	16 / M	Castleman disease hyaline vascular type	Before	**Oral biopsy**: Intraepidermal acantholysis and lichenoid features	**DIF**: C3 and IgG at BMZ, C3 and IgG ICS<br>**IIF**: positive on ME and RBE<br>**Serum panel**: NR<br>**ELISA**: Dsg3<br>**IB**: Plectin<br>**IP**: A2ML1, DSP1, DSP2
	1	8 / M	Castleman disease hyaline vascular type	Before	**Biopsy**: NR	**DIF**: negative<br>**IIF**: positive on ME and RBE<br>**Serum panel**: NR<br>**ELISA**: Dsg3<br>**IB**: Plectin<br>**IP**: A2ML1, DSP1, DSP2
	1	14 / M	Castleman disease plasma cell type	Before	**Biopsy**: NR	**DIF**: negative<br>**IIF**: positive on ME and RBE<br>**Serum panel**: NR<br>**ELISA**: Dsg3<br>**IB**: Plectin<br>**IP**: A2ML1, DSP1, DSP2
	1	10 / F	Castleman disease hyaline vascular type	Before	**Oral biopsy**: lichenoid features	**DIF**: negative<br>**IIF**: positive on ME and RBE<br>**Serum panel**: NR<br>**ELISA**: Dsg3<br>**IB**: Plectin

							**IP**: A2ML1, DSP1, DSP2
**Leyn 2001 (Leyn & Degreef, 2001), Belgium**	Case report	1	76 / F	Mediastinal Thymoma	After	**Oral biopsy**: acantholysis, apoptotic keratinocytes, and Interface dermatitis	**DIF**: IgG at BMZ, IgG ICS<br>**IIF**: positive<br>**Serum panel**: NR<br>**ELISA**: NR<br>**IB**: NR<br>**IP**: NR
**Cordel 2001 (Cordel, Ringeisen, Antoine, Cadranel, & Aractingi, 2001), France**	Case report	1	60 / F	Low-Grade Non-Hodgkin Lymphoma B cell lymphoma type	Before	**Oral biopsy**: Suprabasal acantholysis and necrotic keratinocytes	**DIF**: IgG at BMZ, IgG ICS<br>**IIF**: positive on RBE<br>**Serum panel**: NR<br>**ELISA**: NR<br>**IB**: EVPL<br>**IP**: NR
**Borradori 2001 (Borradori et al., 2001), Switzerland**	Case report	1	61 / F	Grade I Follicular Non-Hodgkin Lymphoma with Bone Marrow and Pleural Metastasis	Before	**Oral biopsy**: necrotic keratinocytes and inflammatory cells infiltration	**DIF**: C3 ICS<br>**IIF**: negative on human skin, ME, and RBE<br>**Serum panel**: NR<br>**ELISA**: NR<br>**IB**: NR<br>**IP**: A2ML1
**Hsiao 2001 (Hsiao, Hsu, Lee, Chen, & Hsieh, 2001), Taiwan**	Case report	1	50 / M	Retroperitoneal Castleman Disease Hyaline Vascular type	Before	**Oral biopsy**: Suprabasal acantholysis	**DIF**: C3 at BMZ, IgG ICS<br>**IIF**: positive on human skin and RBE<br>**Serum panel**: ANA<br>**ELISA**: NR<br>**IB**: BP230, DSP1<br>**IP**: NR
**van der Waal 2000 (Van Der Waal et al., 2000), Netherlands**	Case report	1	56 / F	Retroperitoneal Epithelioid Leiomyosarcoma	Before	**Oral biopsy**: Suprabasal acantholysis and inflammatory cells infiltration	**DIF**: linear C3 and IgG at BMZ and IgG ICS<br>**IIF**: positive on ME and RBE<br>**Serum panel**: ANA, ANCA, StrAb<br>**ELISA**: Dsg1, Dsg3<br>**IB**: BP230, Dsg3, DSP1, DSP2, EVPL, PPL<br>**IP**: A2ML1, Dsg3, EVPL, PPL

**Williams 2000 (Williams, Marks Jr, & Billingsley, 2000), US**	Case report	1	40 / F	Chronic lymphocytic leukaemia	After	**Skin biopsy**: Intraepidermal acantholysis	**DIF**: NR<br>**IIF**: positive on ME<br>**Serum panel**: NR<br>**ELISA**: NR<br>**IB**: NR<br>**IP**: A2ML1, BP230, DPS1, DSP2, PPL
**Mascaró 1999 (Mascaró Jr et al., 1999), Spain**	Case report	1	35 / M	Pelvic Castleman disease hyaline vascular type	After	**Skin biopsy**: Suprabasal acantholysis, dyskeratosis, and Interface dermatitis	**DIF**: IgG ECS<br>**IIF**: positive on human skin and RBE<br>**Serum panel**: ANA, dsDNA<br>**ELISA**: NR<br>**IB**: NR<br>**IP**: A2ML1, BP230, DPS1, DSP2, PPL
**Nousari 1999 (Nousari, Deterding, et al., 1999), US**	Case series	1	13 / M	Mediastinal Castleman disease hyaline vascular type	Before	**Skin biopsy**: acantholysis	**DIF**: C3 and IgG at BMZ; C3 and IgG ECS<br>**IIF**: positive on ME and RBE<br>**Serum panel**: NR<br>**ELISA**: NR<br>**IB**: NR<br>**IP**: A2ML1, BP230, DPS1, DSP2, PPL
		1	39 / M	Low-Grade B-cell Non-Hodgkin Lymphoma with Recurrence	After	**Skin biopsy**: necrotic keratinocytes	**DIF**: C3 at BMZ; C3 and IgG ECS<br>**IIF**: positive on ME and RBE<br>**Serum panel**: NR<br>**ELISA**: NR<br>**IB**: NR<br>**IP**: DPS1, DSP2, PPL
		1	70 / M	Stage 0 Chronic lymphocytic leukaemia	After	**Oral biopsy**: Intraepidermal acantholysis and lichenoid infiltrate	**DIF**: C3 and IgG ECS<br>**IIF**: positive on ME and RBE<br>**Serum panel**: NR<br>**ELISA**: NR

							**IB**: NR<br>**IP**: A2ML1, BP230, DPS1, DSP2, PPL
**Wolff 1999 (Wolff et al., 1999),<br>Germany**	Case report	1	42 / F	Abdominal Castleman disease stroma rich type	Before	**Oral biopsy**: cytoid bodies, inflammatory cells infiltration, and vacuolar degeneration	**DIF**: C3, fibrinogen, and IgM ICS<br>**IIF**: negative on ME and RBE<br>**Serum panel**: ANA, dsDNA<br>**ELISA**: NR<br>**IB**: NR<br>**IP**: A2ML1, BP230, DPS1, DSP2, PPL
**Favia 1998 (Favia, Di Alberti, & Piattelli, 1998),<br>Italy**	Case series	1	48 / F	Low Grade B cell lymphoma with Lung Metastasis	After	**Oral biopsy**: Intraepidermal acantholysis	**DIF**: C3 and IgG at BMZ; C3 and IgG ICS<br>**IIF**: positive on RBE<br>**Serum panel**: NR<br>**ELISA**: NR<br>**IB**: NR<br>**IP**: BP230, DPS1, DSP2, PPL
		1	59 / F	Mediastinal Anaplastic Thymoma with Recurrence	After	**Oral biopsy**: Intraepidermal acantholysis	**DIF**: C3 BMZ; C3 ECS<br>**IIF**: positive on RBE<br>**Serum panel**: NR<br>**ELISA**: NR<br>**IB**: NR<br>**IP**: BP230, DPS1, DSP2, PPL
**van der Waal 1998 (Van Der Waal et al., 1998),**	Case report	1	75 / M	Stage III Non-Hodgkin Lymphoma	Before	**Skin biopsy**: Interface dermatitis with band-like inflammatory infiltrate in the	**DIF**: linear C3 and IgG at BMZ and IgG ICS

**Netherlands**				Diffuse large B-cell lymphoma type		superficial dermis, apoptotic keratinocytes, and vacuolar degeneration	**IIF**: positive on ME and RBE<br>**Serum panel**: NR<br>**ELISA**: Dsg3<br>**IB**: A2ML1, BP230, Dsg3, DSP1, DSP2, EVPL, PPL<br>**IP**: A2ML1, BP230, DSP1, DSP2, EVPL, PPL
**Becker 1998 (Becker et al., 1998), Germany**	Case report	1	84 / M	Waldenström macroglobulinemia	After	**Skin biopsy**: Suprabasal acantholysis, inflammatory cells infiltration, parakeratosis, and vacuolar degeneration	**DIF**: C3 and IgG ICS<br>**IIF**: positive on RBE<br>**Serum panel**: NR<br>**ELISA**: NR<br>**IB**: NR<br>**IP**: NR
**Sapadin 1998 (Sapadin & Anhalt, 1998), US**	Case report	1	54 / M	Chronic lymphocytic leukaemia	After	**Oral biopsy**: Suprabasal acantholysis and inflammatory cells infiltration	**DIF**: IgG ICS<br>**IIF**: positive on ME and RBE<br>**Serum panel**: NR<br>**ELISA**: NR<br>**IB**: NR<br>**IP**: BP230, DPS1, DPS2, PPL
**Mahler 1998 (Mahler et al., 1998), Germany**	Case report	1	63 / M	Low-Grade Follicular non-Hodgkin lymphoma	Before	**Oral biopsy**: erosions and inflammatory cells infiltration	**DIF**: C3 at BMZ and cytoid bodies<br>**IIF**: positive on ME and RBE<br>**Serum panel**: ANA, dsDNA, RNP/Sm complex, Ro<br>**ELISA**: NR<br>**IB**: NR<br>**IP**: A2ML1, EVPL, PPL
**Schoen 1998 (Schoen et al., 1998), Austria**	Case report	1	16 / M	Retroclavicular Inflammatory myofibroblastic tumour	Before	**Oral biopsy**: Suprabasal acantholysis, necrotic keratinocytes, and vacuolar Inteface dermatitis	**DIF**: C3 and IgG at BMZ; C3 and IgG ICS<br>**IIF**: positive on ME and RBE<br>**Serum panel**: NR<br>**ELISA**: NR<br>**IB**: EVPL, PPL

							**IP**: BP230, DSP1, DSP2, PPL
**Kim 1998 (S. C. Kim et al., 1998), Korea**	Case report	1	19 / F	Retroperitoneal Castleman disease hyaline vascular type	Before	**Oral biopsy**: Suprabasal acantholysis, dyskeratosis, and vacuolar degeneration	**DIF**: linear C3 BMZ; C3 and IgG ECS<br>**IIF**: positive on human skin and RBE<br>**Serum panel**: CGs<br>**ELISA**: NR<br>**IB**: EVPL, PPL<br>**IP**: A2ML1, BP230, DPS1, DSP2, PPL
**Bialy-Golan 1996 (Bialy-Golan, Brenner, & Anhalt, 1996), Israel**	Case report	1	50 / M	Non-Hodgkin Lymphoma with Recurrence	After	**Oral biopsy**: Suprabasal acantholysis, dyskeratosis, and vacuolar Interface dermatitis	**DIF**: linear C3 at BMZ; IgG ICS<br>**IIF**: positive on RBE<br>**Serum panel**: NR<br>**ELISA**: NR<br>**IB**: NR<br>**IP**: NR
**Tankel 1993 (Tankel, Tannenbaum, & Parekh, 1993), US**	Case report	1	38 / F	Retroperitonea Well Differentiated Lymphoma	Before	**Skin biopsy**: Suprabasal acantholysis, dyskeratosis, inflammatory cells infiltration, and spongiosis	**DIF**: C3 and IgG ICS<br>**IIF**: positive on human skin, ME, and RBE<br>**Serum panel**: NR<br>**ELISA**: NR<br>**IB**: NR<br>**IP**: A2ML1, BP230, DPS1, DPS2, PPL
**Helm 1993 (Helm, Camisa, Valenzuela, & Allen, 1993), US**	Case report	1	67 / F	Non-Hodgkin Lymphoma Follicular small cleaved cell lymphoma type with Recurrence	After	**Oral biopsy**: Intraepidermal and Subepidermal acantholysis, inflammatory cells infiltration, and lichenoid mucositis	**DIF**: C3 at BMZ; IgG ICS<br>**IIF**: positive on RBE<br>**Serum panel**: NR<br>**ELISA**: NR<br>**IB**: NR<br>**IP**: NR

**Camisa 1992 (Camisa et al., 1992),**<br>**US**	Case series	1	69 / M	Chronic lympho-cytic leukaemia	After	**Skin biopsy**: Suprabasal acantholysis	**DIF**: C3 and IgG ICS<br>**IIF**: positive on RBE<br>**Serum panel**: NR<br>**ELISA**: NR<br>**IB**: NR<br>**IP**: BP230, DPS1, DPS2, PPL
		1	58 / F	Non-Hodgkin Lym-phoma Follicular small cleaved cell lymphoma type	Before	**Extraoral mucosa biopsy**: Suprabasal acantholysis	**DIF**: diffuse fibrinogen and globular C1, IgA, IgG, and IgM at BMZ; C1, C3, and IgG ICS<br>**IIF**: positive on ME and RBE<br>**Serum panel**: NR<br>**ELISA**: NR<br>**IB**: NR<br>**IP**: BP230, DPS1, DPS2, PPL
		1	56 / M	Non-Hodgkin Lym-phoma Diffuse large B-cell lym-phoma type	After	**Oral biopsy**: Intraepidermal and Subepi-dermal acantholysis and inflammatory cells infiltration	**DIF**: linear fibrinogen and IgM at BMZ<br>**IIF**: positive on RBE<br>**Serum panel**: NR<br>**ELISA**: NR<br>**IB**: NR<br>**IP**: BP230, DPS1, DPS2, PPL
**Lam 1992 (Lam et al., 1992),**<br>**US**	Case report	1	77 / M	Non-small cell Lung cancer Squamous cell carcinoma type	Before	**Skin biopsy**: Suprabasal acantholysis and inflammatory cells infiltration	**DIF**: C3, IgA, and IgG ICS<br>**IIF**: positive on ME<br>**Serum panel**: NR<br>**ELISA**: NR<br>**IB**: NR<br>**IP**: NR

Serology

α-SMA: alpha-smooth muscle actin; A2ML1: Alpha-2-macroglobulin-like 1; ACL: Anti-cardiolipin antibodies; AFP: Alpha-fetoprotein; ANA: Anti-nuclear antibodies; BP230: Bullous pemphigoid antigen I; CGs: Cryoglobulins; Dsc1: Desmocollin-1; Dsc2: Desmocollin-2; Dsc3: Desmocollin-3; DIF: Direct Immunofluorescence; dsDNA: Double stranded DNA antibodies; Dsg1: Desmoglein-1; Dsg3: Desmoglein-3; DSP: Desmoplakin; DSP1: Desmoplakin 1; DSP2: Desmoplakin 2; ELISA: Enzyme-Linked Immunosorbent Assay; EPPK1: Epiplakin 1; EVPL: Envoplakin; IB: Immunoblotting; IIF:Indirect immunofluorescence; IP: Immunoprecipitation; PIVKA-II: protein induced by vitamin K absence-II; PPL: Periplakin; PSA: Prostate specific antigen; RNP: Anti-nuclear ribonucleoprotein; Sm: Anti-Smith antibodies; SSA: Sjögren's-syndrome-related antigen A autoantibodies;
